# Baduanjin combined with computerized cognitive remediation therapy for the treatment of schizophrenia : a 8-week randomized controlled trial

**DOI:** 10.1186/s12888-026-08317-1

**Published:** 2026-06-21

**Authors:** Qingjing Xue, Wei Liang, Ling Xiao, Qing Hao, Wenjing Ma, Ziwen Zhou

**Affiliations:** 1 Nursing Department, The Second People’s Hospital of Chuzhou, Chuzhou, Anhui Province China; 2 School of Nursing, Bengbu Medical University, Bengbu, Anhui Province China

**Keywords:** Schizophrenia, Baduanjin, Computerized cognitive remediation therapy

## Abstract

**Background:**

Schizophrenia is a severe mental disorder characterized by complex clinical presentations and marked impairments in social and occupational functioning. While antipsychotic medications are effective in managing positive symptoms, their efficacy in addressing negative symptoms, cognitive deficits, and overall improvements in social functioning and quality of life remains substantially limited.This study aims to evaluate the effects of Baduanjin combined with Computerized Cognitive Remediation Therapy (CCRT) on patients with schizophrenia, thereby contributing to the evidence base for its clinical management.

**Methods:**

A total of 120 hospitalized patients with schizophrenia (aged 29–64 years; 66.67% male) were recruited and randomly allocated to either an intervention group (*n* = 60) or a control group (*n* = 60). Participants in the intervention group received a combined 85-minute session (45 min of CCRT plus 40 min of Baduanjin training) five times per week, whereas the control group received CCRT alone for the same duration. Assessments of psychiatric symptoms, cognitive function, social functioning, and quality of life were conducted at baseline and post-intervention.

**Results:**

Baseline characteristics were comparable between the two groups for all outcome measures (*P* > 0.05). Following the 8-week intervention, within-group analyses showed significant improvements from baseline in The Neurobehavioral Cognitive Status Examination(NCSE), The Scale of Social Function in Psychosis Inpatients( SSPI), and The Schizophrenia Quality of Life Scale(SQLS) scores for both groups (all *P* < 0.05). In contrast, a statistically significant reduction in The Positive and Negative Syndrome Scale(PANSS) total scores was only observed in the intervention group (*P* < 0.05). Moreover, intergroup analysis revealed that the intervention group demonstrated significantly greater improvements across all outcome measures (NCSE, SSPI, SQLS, and PANSS) compared to the control group post-intervention (all *P* < 0.05).

**Conclusion:**

Patients with schizophrenia showed significantly better overall efficacy with an 8-week combined treatment of Baduanjin and CCRT compared to CCRT alone, particularly in improving psychiatric symptoms (especially negative symptoms), enhancing cognitive function, restoring social functioning, and improving quality of life.

**Clinical trial registration information:**

The study protocol of this investigator-initiated randomised controlled trial was formally registered with the ISRCTN registry (registration number: ISRCTN14037337) on 12 May 2026.

## Background

Schizophrenia, a severe mental disorder, is characterized by core symptoms, which often include significant cognitive impairment and social withdrawal [1]. Although antipsychotic medications have achieved breakthroughs in controlling positive symptoms, interventions focused on negative symptoms (e.g., emotional blunting, lack of motivation) and cognitive deficits remain challenging [2]. In recent years, non - pharmacological treatment modalities have increasingly served as crucial adjuncts for improving patient outcomes [3].The Eight Brocades exercise regimen, originating from traditional Chinese medicine and characterized by its relaxed, tranquil, natural, gentle, and slow movement patterns, has demonstrated unique advantages in improving physical function, sleep quality, and emotional state among schizophrenia patients [4]. However, exercise intervention alone has limited effects on deep cognitive restructuring. Meanwhile, CCRT—an evidence-based neuropsychological rehabilitation technique—precisely targets core cognitive domains such as attention, working memory, and executive function via human-computer interaction, and has been proven effective in enhancing patients’ social adaptation abilities [5–7]. The combination of Bajianjin and CCRT aims to establish a comprehensive rehabilitation system addressing both mind and body. This model employs CCRT for intensive cognitive neuroplasticity while incorporating Bajianjin’s mind-body regulation, potentially advancing from“cognitive improvement”to “enhanced quality of life.”This paper examines the potential effectiveness of this combined therapy in improving psychotic symptoms, self-functioning, and overall quality of life in schizophrenia patients.

## Methods

### Subjects recruitment and sample size calculation

All subjects were recruited from Chuzhou Second People’s Hospital and provided informed consent before intervention initiation. Subjects were consecutively numbered by their initials and randomly assigned to one of two groups: the Baduanjin plus CCRT group or the CCRT-only group, using a random number table. Sample size was calculated using G*Power (version 3.1) [8] with the following assumptions: a medium effect size (*f* = 0.25), α = 0.05, power (1-*β*) = 0.80, and a two-way repeated measures ANOVA design. This calculation yielded a required sample size of 54 participants per group. Considering a 10% dropout rate, we aimed to recruit 60 participants per group, resulting in a total sample size of 120 participants.

### Inclusion and exclusion criteria

Inclusion criteria: (1) Diagnosed with schizophrenia by a clinician according to the diagnostic criteria for schizophrenia in the WHO International Classification of Diseases, 10th Revision (ICD-10) [9]; (2) Hospitalized for ≥ 1 year; (3) Maintained stable medication dosage for ≥ 3 months.

Exclusion criteria: (1) Individuals with severe physical conditions, such as serious cardiovascular, pulmonary, or musculoskeletal disorders, that prevent participation in exercise; (2) visual and/or hearing impairments that prevent completion of neurocognitive testing; (3) coexisting mental or neurological disorders.

### Intervention

**Control group**: During an 8-week CCRT treatment cycle, patients received 5 sessions per week, each lasting 45 min, and underwent cognitive training via a local area network (LAN). The intervention included five core modules: attention, working memory, problem-solving, cognitive flexibility, and social cognition. Each module contained 4–6 training tasks, totaling 30 exercises. All training tasks were structured with progressive difficulty and gradually increasing speed, comprising 4–96 corrective trials per exercise. Prior to the main intervention, research team members provided subjects with basic computer operation training. Following the guidance of rehabilitation therapists, subjects engaged in the assigned module exercises. The software adaptively selected appropriate game modules and training difficulty levels based on the subjects’performance, gradually increasing task complexity to guide progressive therapeutic task completion.

**Intervention group**: The research group combined CCRT intervention with Baduanjin exercises. Training was conducted according to the National Sports Administration’s guidelines on Baduanjin movement essentials [10]. The regimen included a 5-minute warm-up, 30 min of Baduanjin practice, and a 5-minute cool-down. Professionally trained and certified nursing staff led patients through the Baduanjin exercises. The intervention cycle lasted 8 weeks, with 5 sessions per week, each lasting 40 min.

### Evaluation tools

#### Psychiatric symptoms

The Positive and Negative Syndrome Scale (PANSS) [11] was used to assess patients’ psychiatric symptoms. This scale stands as one of the most fundamental tools in psychiatric clinical evaluations of schizophrenia, demonstrating robust reliability and validity. Through semi-structured interviews, it quantifies symptom severity and is widely employed for drug efficacy assessment, condition monitoring, and clinical trials.

#### Cognitive function

Cognitive Function: The Neurobehavioral Cognitive Status Examination (NCSE) [12] categorizes cognitive function into five primary domains and independently assesses level of consciousness. This structure enables rapid identification of intact domains while providing detailed evaluation of impaired areas.

#### Social function

The Scale of Social Function in Psychosis Inpatients (SSPI) [13] was used to evaluate patients’ practical abilities in daily living, interpersonal interactions, and social adaptation. It is one of the most widely used social function assessment tools in current domestic psychiatric research.

#### Quality of life

The Schizophrenia Quality of Life Scale (SQLS) [14] is a self-report instrument specifically designed for individuals with schizophrenia. Developed by Wilkinson et al. at the University of Liverpool in the UK, it has been translated and revised by Chinese scholars. It has strong psychometric properties, with a test-retest reliability coefficient of 0.87 and internal consistency alpha coefficients ranging from 0.70 to 0.92. The scale is widely used in clinical research in China.

Group Allocation and Intervention: Patients were randomly allocated to the groups. Due to the nature of the Baduanjin intervention, participants and therapists could not be blinded to group assignment. However, the outcome assessors and statisticians were blinded to group allocation throughout the study period.To prevent potential cross-contamination, all interventions were conducted at the rehabilitation physical therapy center, with the intervention and control groups undergoing their respective treatments in separate batches.

#### Statistical analyses

Statistical analyses were performed using SPSS version 26.0. Quantitative variables following a normal distribution are reported as mean ± standard deviation (‾**X** ± s), while categorical data are presented as n (%). For baseline characteristics, independent samples t-tests or chi-square tests were used for quantitative or categorical data, respectively (with Fisher’s exact test applied when *n* < 5). Outcome measures were analyzed using two-way repeated measures ANOVA, with specific comparisons between subscales within PANSS and SSPI performed using t-test. *P* < 0.05 was considered statistically significant.

## Results

### Study population and baseline characteristics

**Fig. 1:**
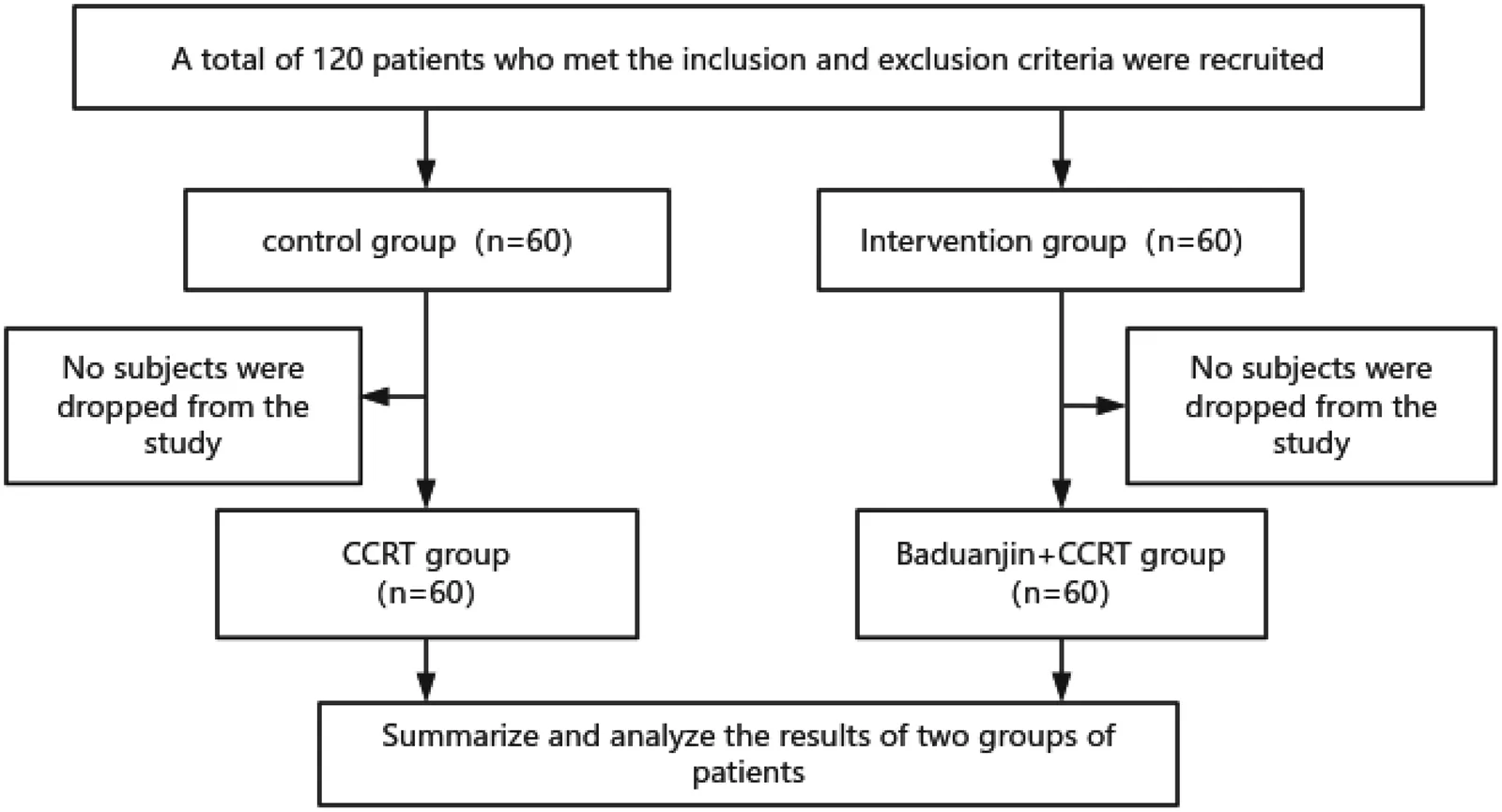
The flow of participants

Between June 2024 and October 2025, 120 patients with schizophrenia were recruited from Chuzhou Second People’s Hospital. All subjects completed both assessments and interventions. The participant flow is presented in Fig. 1 and baseline characteristics are summarized in Table 1. As shown in Table 1, no statistically significant differences were observed between the two groups in terms of age, educational attainment, disease duration, or other demographic and clinical variables (*P* > 0.05).

**Table 1:** Demographic characteristics and medication use of participants

group	Intervention Group (*n* = 60)	Control Group (*n* = 60)	T/χ^2^	DF	*P* value
Age (year)	51.53 ± 8.41	49.48 ± 8.45	1.332	118	0.186
Education (year)			0.592	4	0.964
Primary School Dropout	7(11.67)	9(15.00)			
Elementary school	23(38.30)	23(38.30)			
junior high school	21(35.00)	18(30.00)			
high school	4(6.67)	5(8.33)			
college	5(8.33)	5(8.33)			
Duration of illness (year)	24.05 ± 10.80	20.82 ± 9.58	1.735	118	0.085
Gender			0.150	1	0.699
male	41(68.33)	39(65.00)			
female	19(31.67)	21(35.00)			
Marital status			3.013	3	0.390
Unmarried	35(58.33)	27(45.00)			
Married	10(16.67)	11(18.33)			
Widowed	3(5.00)	7(11.67)			
Medication			0.000	1	1.000
AP typical	1(1.67)	1(1.67)			
Atypical	59(98.33)	59(98.33)			
Total CPZ equivalent dosages (g)	311.25 ± 181.41	290.08 ± 161.11	0.676	118	0.501

Note: CPZ, chlorpromazine; AP, Antipsychotic; PANSS, Positive and Negative Syndrome Scale.

### Two-way repeated measures ANOVA test results

Two-way repeated measures ANOVA demonstrated significant effects of the intervention on mental state, social functioning, cognitive function, and quality of life. The “group × time” interaction was significant for all outcome measures: PANSS (F = 4.958, *P* = 0.028), SSPI (F = 108.45, *P* < 0.001), NCSE (F = 9.360, *P* = 0.003), and SQLS (F = 24.147, *P* < 0.001). Post-hoc analyses revealed that the intervention group showed significant improvements in all outcome measures compared to baseline (all *P* < 0.05) and demonstrated superior results compared to the concurrent control group (all *P* < 0.05) (Table 2).

**Table 2:** Two-Way Repeated-Measures ANOVA for psychiatric symptoms, social function, cognitive function and quality of life (´χ± s)

Group	PANSS	SSPI	NCSE	SQLS
Control Group (*n* = 60)				
Baseline	63.23 ± 10.03	14.45 ± 6.33	55.05 ± 9.90	18.23 ± 2.89
After intervention	63.48 ± 9.53	16.27 ± 6.02^a^	56.75 ± 10.68^a^	19.87 ± 2.67 ^a^
Change (Δ)	0.25 ± 0.96	1.82 ± 6.31	1.17 ± 0.72	1.64 ± 1.45
Intervention Group (*n* = 60)				
Baseline	63.37 ± 9.49	14.50 ± 7.53	56.59 ± 5.56	17.95 ± 3.12
After intervention	60.58 ± 8.75^ab^	25.62 ± 7.00^ab^	61.41 ± 4.67^ab^	21.56 ± 2.89 ^ab^
Change (Δ)	2.78 ± 0.96	11.12 ± 0.63	4.82 ± 0.72	+ 3.61 ± 1.78
Group × Time Interaction				
F-value	4.958	108.45	9.360	24.147
*ŋ2*	0.04	0.479	0.073	0.170
*P*-value	0.028	< 0.001	0.003	< 0.001

^a P < 0.05, compared with baseline within the same group (based on the simple effects analysis of the repeated-measures ANOVA).^

^b P < 0.05, compared with the control group at the same time point (based on the simple effects analysis of the repeated-measures ANOVA).^

^The P-values in the "Group × Time Interaction" row are derived from the two-way repeated-measures ANOVA and indicate that the improvement trajectory was significantly different between the two groups over time.^

### Results of the T-test

Results indicated significant improvements in negative symptoms and the total PANSS score in the intervention group post-intervention. Additionally, although both groups showed enhanced performance in self-care, initiative/engagement, and social activity skills on the SSPI from baseline to post-intervention, the intervention group demonstrated greater improvement than the control group (all *P* < 0.001). These findings are detailed in Tables 3 and 4.

**Table 3:** T-test for psychiatric symptoms

Group	Positive symptom	Negative symptom	General pathology	Totals
Control group (*n* = 60)				
Baseline	11.87 ± 4.49	23.22 ± 7.82	28.15 ± 5.94	63.23 ± 10.03
After intervention	11.43 ± 4.38	23.68 ± 7.70	28.3 ± 5.76	63.48 ± 9.53
Intervention group (*n* = 60)				
Baseline	11.42 ± 4.40	23.58 ± 7.61	28.37 ± 5.76	63.37 ± 9.49
After intervention	10.78 ± 3.97	20.87 ± 5.80^ab^	28.93 ± 5.62	60.58 ± 8.75^ab^
*P* value	0.699	< 0.001	0.677	0.029

^aThe comparison between the two groups after treatment and before treatment, P < 0.05^

^b Compare the degree of improvement between two groups of control and intervention groups, P < 0.05;^

^The P-value is calculated by comparing the degree of improvement between the intervention group and the control group after treatment.^

**Table 4:** T-test for social function

Group	The daily living skills	Motivation and socialization	Sociamobility skills	Totals
Control group (*n* = 60)				
Baseline	5.42 ± 1.89	6.03 ± 3.23	3.00 ± 2.07	18.23 ± 2.89
After intervention	5.95 ± 1.84^a^	6.78 ± 3.00^a^	3.53 ± 2.08^a^	19.87 ± 2.67 ^a^
Intervention group (*n* = 60)				
Baseline	5.58 ± 2.12	5.85 ± 3.59	3.067 ± 2.59	14.50 ± 7.53
After intervention	8.32 ± 1.60^ab^	10.68 ± 3.48^ab^	6.62 ± 2.97^ab^	25.62 ± 7.00^ab^
*P* value	< 0.001	< 0.001	< 0.001	< 0.001

^a^The comparison between the two groups after treatment and before treatment, P < 0.05

^b^ Compare the degree of improvement between two groups of control and intervention groups, P < 0.05;

The P-value is calculated by comparing the degree of improvement between the intervention group and the control group after treatment.

## Discussion

Schizophrenia is a severe mental disorder predominantly affecting young adults and working-age populations, with its etiology remaining largely unknown. Characterized by a chronic and relapsing course [15, 16], it imposes a significant burden on patients and their families. With increased health awareness and improved medical care, treatment for chronic schizophrenia now extends beyond symptom management, generating new demands for rehabilitation outcomes that maximize quality of life, restore social functioning, and alleviate illness-related stress.

The current study demonstrated significantly superior efficacy of the combined intervention in improving overall symptoms of schizophrenia, particularly negative symptoms, compared to the control group, consistent with prior findings by Gao et al. [17]. Although the present study did not directly measure neurobiological parameters, previous literature suggests potential mechanisms that may explain this synergistic effect. For instance, Baduanjin—as a slow, continuous, and attention-demanding aerobic exercise—could theoretically improve motivational deficits and emotional blunting, key components of negative symptoms, by modulating central neurotransmitter systems (e.g., serotonergic activity) [18]. Second, structured movement practice may serve as a form of behavioral activation, helping counteract psychomotor retardation and difficulties in initiating daily activities. Third, the guided group practice format provides a structured, low‑pressure social interaction environment, potentially mitigating social withdrawal tendencies [18]. Thus, based on existing literature, we hypothesize that Baduanjin exerts a comprehensive effect on core negative symptom dimensions not through a single mechanism, but simultaneously across neurobiological, behavioral activation, and psychosocial pathways. These interpretations remain speculative and require empirical validation in future studies.

This study also demonstrates that CCRT effectively enhances cognitive function in schizophrenia patients, consistent with the findings of Ma et al. [19–21]. The proposed mechanism, as suggested in prior research, centers on neuroplasticity and cognitive remodeling, whereby CCRT systematically induces functional remodeling of neural circuits through repetitive cognitive task training. This may enhance the brain’s processing efficiency for complex cognitive tasks by altering low‑frequency amplitude (ALFF) in key regions such as the prefrontal cortex and anterior cingulate cortex [22]. Notably, the combined intervention group showed greater cognitive improvement than the CCRT‑alone group. A possible explanation, derived from previous reports, is that Baduanjin—a mind‑body exercise integrating specific breathing regulation and dynamic stretching—promotes peripheral and cerebral blood circulation. By enhancing cerebral oxygen supply and optimizing physiological activation of the cerebral cortex, Baduanjin could potentially provide a more responsive neural foundation for cognitive task execution [23]. Second, studies indicate that Baduanjin practice modulates functional connectivity in brain networks supporting higher‑order cognitive control. Specifically, it may enhance regulatory functions in the dorsolateral prefrontal cortex (DLPFC) and alter resting‑state activity in the default mode network (DMN), thereby supporting executive function and other core cognitive domains [24]. Additionally, Baduanjin may reduce psychological stress by balancing autonomic nervous system activity and mitigating stress responses [24], which could free up cognitive resources and further enhance cognitive performance. In summary, the observed synergistic effects might be explained by multiple complementary mechanisms: Baduanjin improving the overall mind‑body state and optimizing the brain’s “hardware” environment, while CCRT targets “software”‑level neurocognitive remodeling. However, as none of these neurobiological or hemodynamic variables were directly measured in our study, these explanations remain hypothetical.

Regarding social functioning, both the combined intervention group and the CCRT‑only control group showed significant improvements, but the combined group demonstrated greater improvement. Based on existing literature, this synergistic effect likely arises from the complementary roles of both interventions across different dimensions of social functioning. CCRT employs targeted training to enhance patients’ social cognitive abilities—including emotion recognition and interpretation of social cues—thereby enabling the construction of more accurate cognitive representations for social interactions [25]. Baduanjin, through its mind‑body regulation, may utilize its unique breathing‑movement coordination pattern to help regulate neuroendocrine homeostasis (e.g., reducing stress‑related cortisol levels) and improve foundational behavioral dimensions related to social motivation, such as personal neatness, mental alertness, and emotional expression [26, 27]. According to prior hypotheses, this combination represents more than a simple summation; it suggests a deep integration at the mechanistic level between “social cognitive reconstruction” and “social behavioral activation,” which could effectively promote overall restoration of social functioning. Again, these mechanistic interpretations are speculative and await direct testing in future research.

Individuals with schizophrenia often experience deficits in attention, memory, and executive functions (e.g., planning). CCRT employs high‑intensity cognitive training (e.g., working memory exercises) to enhance neural efficiency and promote brain plasticity, thereby alleviating negative symptoms and improving overall quality of life [28]. Additionally, CCRT may enhance cognitive potential through targeted brain training [29]. Baduanjin, a gentle traditional Chinese exercise, has been reported to improve physiological functions such as balance, logical memory, and social interaction interest, fostering long‑term adherence and high self‑efficacy [30]. Although the present study did not directly assess neural or physiological mediators, it is plausible that this combination synergistically enhances patients’ overall quality of life by addressing both cognitive and physiological aspects, achieving “internal and external cultivation” as suggested in traditional exercise theories. Future studies incorporating biomarkers and neuroimaging are needed to confirm these pathways.

Limitations and future directions

This study has several limitations. First, the 8‑week follow‑up period is insufficient to assess long‑term effects in a chronic condition like schizophrenia. Longer observation periods are needed to determine whether improvements in clinical symptoms, cognitive function, social functioning, and quality of life persist beyond the immediate post‑intervention phase. Some early improvements may diminish over time, while extended interventions could consolidate or even enhance therapeutic effects. Therefore, future studies with extended follow‑up periods of 6 to 12 months are needed to provide stronger evidence regarding the long‑term clinical value of combined therapy. Second, all participants were recruited from a single center (Chuzhou Second People’s Hospital). The lack of a multicenter design may limit the generalizability of our findings and reduce statistical power for detecting more subtle treatment effects. Although significant differences were observed in the primary outcome measures, the external validity of these results requires further validation. Future studies should therefore prioritize multicenter trials with expanded sample sizes. Additionally, employing longer follow‑up periods and predefined thresholds for clinically meaningful change is crucial for determining the true clinical impact of observed alterations. Third, as noted above, the mechanistic explanations proposed in this discussion are based on previous literature and were not directly tested in our study. Future research should directly measure candidate biomarkers (e.g., serum BDNF, inflammatory cytokines, or functional neuroimaging parameters) to empirically validate the hypothesized pathways.

## Conclusion

The findings indicate that an 8‑week combined intervention of Baduanjin and CCRT significantly improved psychotic symptoms, cognitive function, social functioning, and quality of life in schizophrenia patients more effectively than CCRT alone. Based on previous literature, we speculate that this synergy may stem from complementary effects across physiological, molecular, and behavioral domains. For example, Baduanjin has been reported to enhance cerebral blood flow and hippocampal plasticity, potentially supporting cognitive training; CCRT has been associated with increased BDNF levels, while Baduanjin may sustain this elevation through neuroendocrine modulation. Behaviorally, Baduanjin may alleviate negative emotions, improving patient motivation and adherence to CCRT. However, as these neurobiological pathways were not directly assessed in our study, these proposed mechanisms remain hypothetical and require empirical validation in future research. Collectively, the clinical improvements observed in this trial suggest that this multi‑level synergy provides a novel holistic non‑pharmacological approach for schizophrenia rehabilitation. Future studies directly measuring mechanistic variables are warranted to confirm the underlying pathways.

## Data Availability

The datasets used and/or analyzed during the current study are available from the corresponding author on reasonable request.

## Abbreviations

CCRT: Computerized cognitive remediation therapy
RCT: Randomized controlled trial
ICD-10: International statistical classification of diseases and related health problems 10th revision
PANSS: The positive and negative syndrome scale
NCSE: The neurobehavioral cognitive status examination
SSPI: The scale of social function in psychosis inpatients
SQLS: The schizophrenia quality of life scale
